# Design and Synthesis of NTU-9/C_3_N_4_ Photocatalysts: Effects of NTU-9 Content and Composite Preparation Method

**DOI:** 10.3390/ma16145007

**Published:** 2023-07-14

**Authors:** Damian Makowski, Wojciech Lisowski, Mateusz A. Baluk, Tomasz Klimczuk, Beata Bajorowicz

**Affiliations:** 1 Department of Environmental Technology, Faculty of Chemistry, University of Gdansk, 80-308 Gdansk, Poland; 2 Institute of Physical Chemistry, Polish Academy of Sciences, 01-224 Warsaw, Poland; 3 Department of Solid State Physics, Faculty of Applied Physics and Mathematics, Gdansk University of Technology, 80-233 Gdansk, Poland

**Keywords:** photocatalysts, NTU-9/g-C_3_N_4_, metal–organic frameworks, toluene photodegradation, hybrid materials

## Abstract

Hybrid materials based on graphitic carbon nitride (g-C_3_N_4_) and NTU-9 metal–organic frameworks (MOF) were designed and prepared via solvothermal synthesis and calcination in air. The as-prepared photocatalysts were subsequently characterized using Brunauer–Emmett–Teller (BET) analysis, UV-Vis diffuse reflectance spectroscopy (DRS), photoluminescence (PL) emission spectroscopy, X-ray diffraction (XRD), X-ray photoelectron spectroscopy (XPS), and scanning electron microscopy (SEM). The obtained NTU-9/C_3_N_4_ composites showed a greatly improved photocatalytic performance for the degradation of toluene in the gas phase under LED visible-light irradiation (λ_max_ = 415 nm). The physicochemical properties and photocatalytic activities of the obtained NTU-9/C_3_N_4_ materials were tuned by varying the NTU-9 content (5–15 wt%) and preparation method of the composite materials. For composites prepared by calcination, the photocatalytic activity increased with decreasing NTU-9 content as a result of the formation of TiO_2_ from the MOFs. The best photocatalytic performance (65% of toluene was photodegraded after 60 min) was achieved by the NTU-9/C_3_N_4_ sample prepared via the solvothermal method and containing 15 wt% MOF, which can be attributed to the appropriate amount and stable combination of composite components, efficient charge separation, and enhanced visible-light absorption ability. The photocatalytic mechanisms of the prepared hybrid materials depending on the preparation method are also discussed.

## 1. Introduction

Currently, one of the greatest challenges associated with industrial development is the production of large amounts of pollutants that negatively affect all known ecosystems and living organisms, including humans. The industrial branches responsible for a significant proportion of harmful substances entering the environment include the agricultural, dyeing, electronics, transport, energy, and paper industries [1]. Particular attention is therefore being paid to the development of technologies that can effectively reduce and remove pollutants from the environment [2,3,4,5,6]. Photocatalysis is becoming increasingly important for pollutant degradation, the application of which involves the use of photocatalysts [7,8,9,10]. The most desirable photocatalysts are those that are active under visible light and achieve satisfactory photocatalytic reaction yields. 

A highly promising material that can be applied as a photocatalyst is graphitic carbon nitride (g-C_3_N_4_), which has an energy bandgap of approximately 2.7 eV [11,12]. It is widely used because of its low toxicity, low cost, ease of production, and physicochemical stability. However, despite the advantages of g-C_3_N_4_, its photocatalytic activity must be improved to obtain high photocatalytic reaction yields under visible-light irradiation. One method for enhancing the photocatalytic activity of g-C_3_N_4_ is to create composites using materials such as quantum dots (QDs) [13,14,15] or nanoparticles (NPs) [16,17,18,19,20]. Recently, the modification of photocatalysts with metal–organic frameworks (MOFs) has gained importance. Huang et al. prepared a composite of g-C_3_N_4_ and MIL-53(Fe) MOFs that was successfully used to reduce Cr^6+^ ions under visible light irradiation [21]. Cao et al. investigated a composite composed of g-C_3_N_4_ and MIL-68(In)-NH_2_ MOFs for the photodegradation of ibuprofen in an aqueous medium [22]. Devarayapalli et al. modified g-C_3_N_4_ with ZIF-67 MOFs, and the resulting composite was used for the photogeneration of hydrogen [23]. The modification of g-C_3_N_4_ with MOFs can also successfully improve the photocatalytic reduction in CO_2_. Dao et al. studied a composite of g-C_3_N_4_ and NH_2_-MIL-101(Fe) and proved that it exhibited better photocatalytic CO_2_ reduction ability than pristine materials [24]. MOFs are used not only as modifying materials, but also as precursors to produce MOF-derived materials with porous structures that provide better photocatalytic properties than MOFs. The most promising examples of metal-oxide-derived MOFs include TiO_2_ [25,26], ZnO [27], and Fe_2_O_3_ [28]. However, despite the progress in obtaining various MOF-based materials for photocatalysis, there is still the need for the further development of novel composites and appropriate preparation strategies to obtain effective photocatalytic materials that can be excited by visible light.

In this regard, herein, we designed and prepared novel photocatalysts based on g-C_3_N_4_ and NTU-9 MOFs with a titanium metal core. The NTU-9/C_3_N_4_ hybrid materials were obtained by two different strategies and the effects of the composite coupling method and MOF content on the morphologies, physicochemical properties, and photocatalytic activities of the composites were systematically investigated. The obtained hybrid materials showed improved activity toward the photodegradation of toluene in the gas phase under visible-light irradiation compared to the starting materials. Based on obtained results, the photocatalytic mechanisms depending on the coupling method of NTU-9 and C_3_N_4_ were proposed for the first time.

## 2. Materials and Methods

### 2.1. Materials

Thiourea (99%, Pol-Aura, Olsztyn, Poland) was used as a precursor for the synthesis of g-C_3_N_4_. For the preparation of NTU-9 MOFs, the following reagents were used: dihydroxyterephthalic acid (97%, Aldrich, St. Louis, MO, USA) as a linker, acetic acid (98%, WarChem, Warszawa, Poland) as a solvent, and titanium isoproxide (95%, Acros Organics, Geel, Belgium) as a metal precursor.

### 2.2. Synthesis of g-C_3_N_4_

The preparation of g-C_3_N_4_ from thiourea was performed by annealing the precursor at a high temperature [29]. Thiourea (10 g) was added to a porcelain crucible with a lid and placed in a muffle furnace. Calcination was performed at 550 °C for 4 h, with the temperature being increased at a rate of 5 °C/min. The resulting g-C_3_N_4_ powder was ground in a mortar to unify the samples.

### 2.3. Synthesis of NTU-9 MOF

NTU-9 was synthesized using a method similar to that described by Kaur et al. [30]. First, 2,5-dihydroxyterephthalic acid (H_4_DOBDC; 0.15 g) was added to a Teflon vessel. Concentrated acetic acid (3 mL) and titanium isopropoxide (0.20 mL) were added to the vessel, resulting in a brick-red mixture being formed. The mixture was then stirred for 15 min. The Teflon vessel was then placed in the reactor, and the reaction was run at 120 °C for 5 days. The resulting precipitate was washed five times with methanol, obtaining a dark red powder after drying.

### 2.4. g-Preparation of g-C_3_N_4_/NTU-9 Composites

Composites of g-C_3_N_4_ and NTU-9 were prepared using two different methods: solvothermal synthesis and calcination in air. Each synthesis method was carried out three times to obtain composites with different contents of MOFs in relation to g-C_3_N_4_: 5, 10, and 15 wt%.

For solvothermal synthesis, 3 mL concentrated acetic acid, 0.15 mL H_4_DOBDC as the linker, 0.20 mL Ti(i-OPr)_4_ as the metal precursor, and an appropriate amount of C_3_N_4_ powder were mixed to obtain composites with 5, 10, and 15 wt% NTU-9. The remaining process was analogous to the synthesis of NTU-9.

For calcination in an air atmosphere, appropriate amounts of NTU-9 and g-C_3_N_4_ were mixed in a mortar. The resulting powder mixture was introduced into a crucible and calcined in a tube furnace under an air atmosphere (300 °C, 3 h, 5 °C/min), obtaining a dark red powder of g-C_3_N_4_/NTU-9 composite.

### 2.5. Characterization

The UV-Vis diffuse reflectance spectra (DRS) of the synthesized materials were measured using a Thermo Scientific Evolution 220 UV-Visible spectrophotometer. UV-VIS spectra were recorded in the 200–800 nm range using barium sulfate as a reference. Photoluminescence measurements were conducted using a Perkin Elmer UV LS 50 B spectrometer. The PL spectra were recorded in the wavelength range of 300–700 nm, and the samples were excited at a wavelength of 315 nm. Powder X-ray diffraction (PXRD; Bruker D2 Phaser diffractometer with a LynxEye-XE detector; Cu Kα radiation λ = 1.54 Å) was used to measure the phase compositions of the samples. Nitrogen adsorption–desorption isotherms were measured using a surface area and pore size analyzer (3P Micro 200, 3P Instruments GmbH & Co. KG, Odelzhausen, Germany). Specific surface areas were calculated using the typical Brunauer–Emmett–Teller (BET) method. The surface elemental compositions of the photocatalysts were evaluated by X-ray photoelectron spectroscopy (XPS) using a PHl 5000 VersaProbeTM Scanning ESCA Microprobe (ULVAC-PHI, Chigasaki, Japan). High-resolution (HR) XPS spectra were recorded using monochromatic Al-Kα radiation (hν = 1486.6 eV) from an X-ray source operating at 25 W and 15 kV, with a 100 µm spot size. The pass energy and energy step size of the analyzer were 23.5 eV and 0.1 eV, respectively. The binding energy scale was referenced to the C 1s peak at BE = 284.8 eV. The transmission function of the spectrometer was determined to quantify the PHI MultiPak sensitivity factors. The morphologies of the obtained materials were determined using a scanning electron microscope (SEM; JEOL JSM-7610F, Tokyo, Japan) operated in the vacuum mode.

### 2.6. Measurement of Photocatalytic Activity in the Gas Phase

Photocatalytic activity measurements in the gas phase were performed using a specially designed system. The photocatalytic setup consisted of a reactor equipped with LEDs emitting irradiation at a wavelength of 415 nm. The photocatalyst was deposited on a glass plate and placed in a stainless-steel reactor with a quartz glass window. An air–toluene mixture at a concentration of 200 ppm was passed through the system for a period of 1 min, after which the inlet and outlet valves of the reactor were closed. The photocatalytic reactor was then maintained in the dark for 30 min to establish an adsorption–desorption equilibrium in the system. Before starting the irradiation, the first reference measurement was performed by collecting a gaseous sample from the reactor using a gas syringe. To determine the toluene concentration, samples were collected every 20 min during 60 min of irradiation. The analysis of toluene concentration in the gas phase was carried out using a Perkin Elmer Clarus 500 GC (Perkin Elmer, Waltham, MA, USA) equipped with a 30 m × 0.25 mm Elite-5 MS capillary column (film thickness: 0.25 μm) and a flame ionization detector (FID).

## 3. Results and Discussion

A series of g-C_3_N_4_-based photocatalysts were prepared by first simply calcinating thiourea, followed by coupling with NTU-9 MOFs using the two above-mentioned synthesis methods. The sample labels, preparation routes, and characteristics of the prepared samples are listed in Table 1.

### 3.1. BET Surface Area

**Figure 1:**
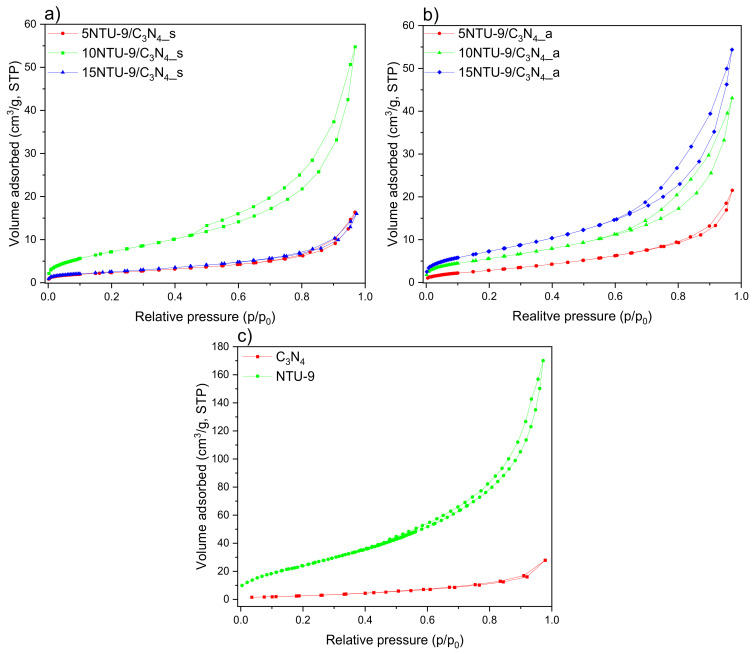
Nitrogen adsorption–desorption isotherms of (**a**) g-C_3_N_4_/NTU-9 obtained via solvothermal synthesis, (**b**) g-C_3_N_4_/NTU-9 prepared by calcination in air, and (**c**) pristine materials.

Nitrogen adsorption–desorption isotherms were used to determine the BET surface areas and porosities of the obtained NTU-9/C_3_N_4_ composites (Table 1, Figure 1). N_2_ sorption data and BET data are of great importance in estimating the surface properties of the tested materials [31,32]. The surface areas of the as-prepared materials ranged over 8–27 m^2^/g, depending on the amount of NTU 9 and the composite preparation method. All obtained BET isotherms are type IV owing to the presence of a hysteresis loop, which indicates that the composites were mesoporous [33,34]. Among the NTU-9/C_3_N_4_ composites obtained by solvothermal method, the largest BET surface was exhibited by the sample with an optimal amount (10 wt%) of NTU-9 MOF in the composites. The modification of C_3_N_4_ with higher amount of NTU-9 MOF (15%) using the solvothermal method revealed a lower surface area, probably due to the influence of two factors: (i) partial blockage of g-C_3_N_4_ pores caused by too high an amount of NTU-9 and/or (ii) the collapse of MOF pores during the solvothermal coupling process. In the case of hybrid materials, NTU-9/C_3_N_4_ obtained by the calcination formation of TiO_2_ as a result of the decomposition of NTU-9 was observed. Therefore, for NTU-9/C_3_N_4_ samples obtained by calcination, a different trend was observed: the specific surface area increases with the increasing content of TiO_2_ derived from MOF in the composites and the samples with the highest amount of TiO_2_ derived from NTU-9 exhibited the largest surface area among all obtained samples. A similar result was reported by Boonprakob et al. [35], who observed that upon increasing the TiO_2_ loading, the surface area increases for g-C_3_N_4_/TiO_2_ composites [35].

### 3.2. Light Absorption Properties

**Figure 2:**
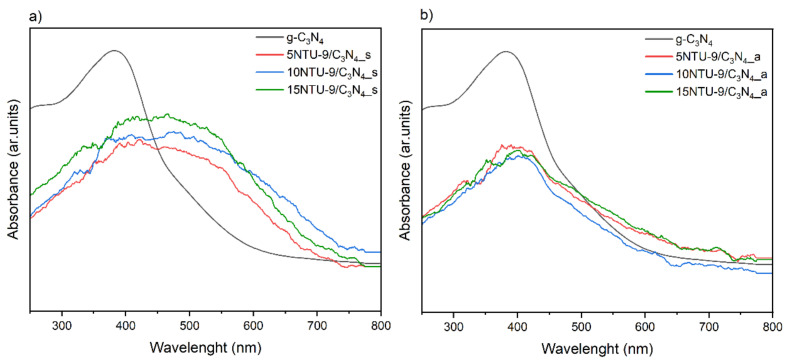
UV-VIS absorption spectra for g-C_3_N_4_/NTU-9 composites obtained by two methods: (**a**) solvothermal synthesis, (**b**) calcination in air.

To evaluate the light absorption capacities of the prepared samples, UV-Vis absorption spectra were measured (Figure 2). The UV-Vis spectrum of g-C_3_N_4_ shows a vivid band with a maximum at approximately 380 nm. This spectrum indicates that the sample absorbs UV-Vis radiation in the wavelength range of 250–550 nm. The DRS UV-Vis spectra (Figure 2) show that both the absorption maxima and absorbed light irradiation ranges for all composites obtained by the solvothermal method are very similar to one another. It can also be observed that the samples with the highest content of the NTU-9 MOF had the highest absorption values. The UV-Vis spectra also showed that the widening of the wavelength range of light that could be absorbed by the composites was a direct result of the presence of NTU-9 in them. For samples obtained by calcination in air, the absorption bands are slightly shifted toward wavelengths in the UV range, but are also apparently less intense than those of g-C_3_N_4_. Therefore, based on the UV-Vis spectra, it can be concluded that the composites obtained by calcination in air showed lower values of visible-range radiation absorption than those obtained by the solvothermal route. In addition, Figure S1 depicts the Tauc plots determined from UV-Vis spectra of g-C_3_N_4_ and NTU-9. The band gap energies calculated for g-C_3_N_4_ and NTU-9 are found to be 2.48 eV and 1.67 eV, respectively.

### 3.3. Photoluminescence Properties

**Figure 3:**
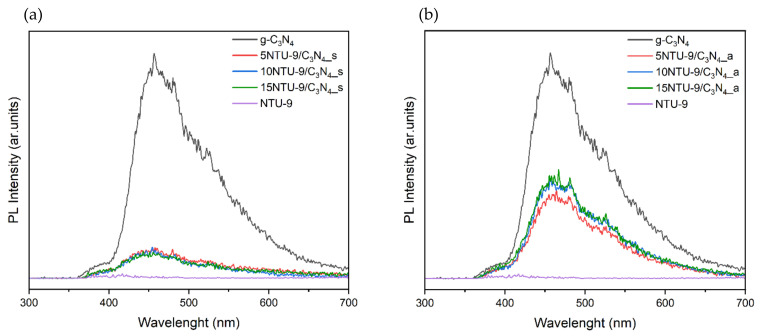
Photoluminescence intensity as a function of wavelength for g-C_3_N_4_/NTU-9 composites obtained by two different methods: (**a**) solvothermal synthesis, (**b**) calcination in air.

PL studies can reflect the recombination frequency of reactive electron–hole pairs formed by the excitation of a photocatalyst [36,37]. The PL spectra for the samples are shown in Figure 3. The PL spectra obtained for the g-C_3_N_4_-based samples showed that all the photocatalysts exhibited maximum PL at approximately 450 nm, which is in good agreement with the data reported by Aleksandrzyk et al. [38] and Ghouri et al. [39]. The PL intensities of the obtained composites (Figure 3) clearly show that the samples obtained by the solvothermal method exhibited the lowest PL intensities, which suggests that these photocatalysts had the lowest recombination frequency of electron–hole pairs among the obtained samples. The highest PL intensities were exhibited by the composites prepared by calcination in air; thus, we can expect the highest recombination of reactive electron–hole pairs. In addition, pristine NTU-9 MOF showed a very low PL intensity, and its presence in the composite composition significantly reduced the PL intensity compared to that of the pristine g-C_3_N_4_ sample. Moreover, it was also observed that PL intensity decreased with increasing amounts of NTU-9 in composites obtained by the solvothermal method (Figure 2b). The effects of different MOFs on lowering the PL intensities of various composite materials has been previously reported in the literature [40,41].

### 3.4. XRD Analysis

**Figure 4:**
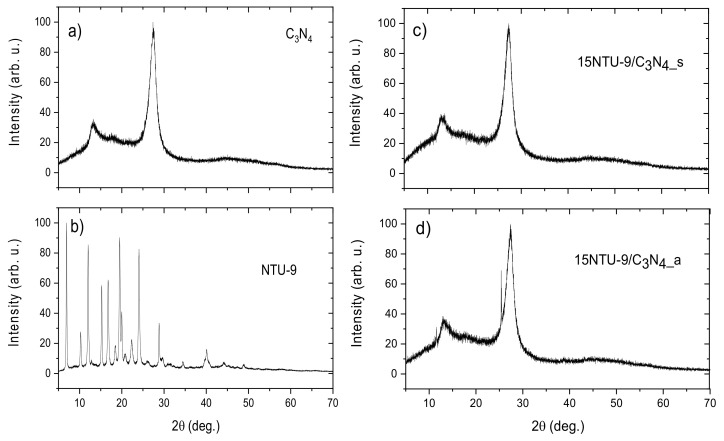
Powder XRD patterns for (**a**) pristine g-C_3_N_4_, (**b**) NTU-9 MOF, (**c**) NTU-9/C_3_N_4_ hybrid obtained by solvothermal method and (**d**) NTU-9/C_3_N_4_ hybrid obtained by the calcination method.

Figure 4 and Figure S2 show the XRD patterns of pristine C_3_N_4_, NTU-9 MOFs, and NTU-9/C_3_N_4_ composite materials obtained via two different methods: solvothermal synthesis and calcination in air. The diffraction pattern of pristine g-C_3_N_4_ (Figure 4a) reveals distinctive peaks located at approximately 27.4° and 13.2°, which can be indexed to the (002) and (100) crystal planes of g-C_3_N_4_, corresponding to the graphite-like stacking of aromatic systems and interlayer structural packing, respectively [42,43]. The diffraction pattern of NTU-9 (Figure 4b) corresponded well with that of a previous report, confirming the successful formation of the desired framework structure [44]. After the modification of C_3_N_4_ with NTU-9 MOFs, the XRD patterns of the resulting hybrid materials were similar to those of pristine g-C_3_N_4_, suggesting a low MOF content and indicating that the coupling method did not destroy the crystal structure of C_3_N_4_. Moreover, for the 15NTU-9/C_3_N_4__a composite (Figure 4d), an additional diffraction peak at approximately 25.3° was observed, suggesting the formation of TiO_2_ [45] from the Ti-based MOFs during calcination in air, which is in good agreement with the XPS and DRS UV-Vis spectra.

### 3.5. XPS Analysis

**Figure 5:**
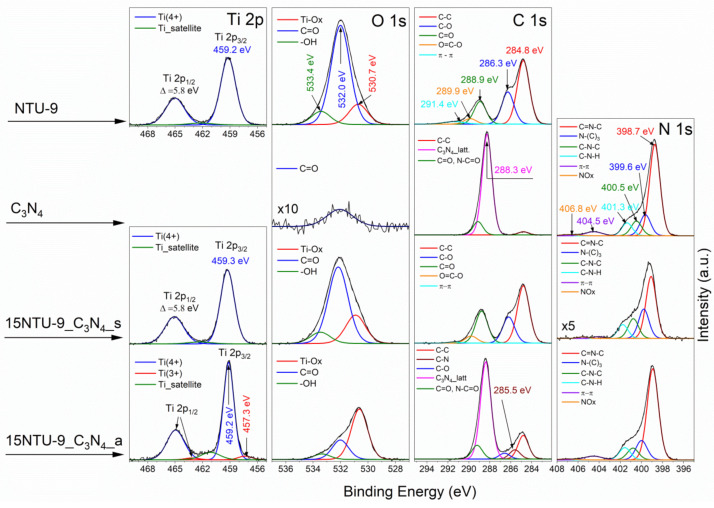
High resolution XPS spectra of elements detected in pristine g-C_3_N_4_, NTU-9 MOF, and NTU-9/C_3_N_4_ hybrid materials.

The surface elemental compositions of the pristine g-C_3_N_4_, NTU-9 MOF, and NTU-9/g-C_3_N_4_ hybrid materials, as evaluated by XPS, are presented in Table 2. The high-resolution (HR) spectra of the elements detected on the surfaces of the pristine materials and selected 15NTU-9/C_3_N_4__s and 15NTU-9/C_3_N_4__a samples are shown in Figure 5. The chemical characteristics of the elements identified after deconvolution of their HR spectra are shown in Figure 5 and Table S1. The presence of titanium, oxygen and carbon in the pristine NTU-9 composite was well characterized by the Ti 2p, O 1s, and C 1s spectra [46]. The Ti 2p spectrum clearly identified the Ti(4+) state of titanium (Ti 2p_3/2_ signal at BE = 459.2 eV) [47]. The HR spectrum of O 1s can be deconvoluted into three peaks, located at 530.7, 532.0, and 533.4 eV, and that of C 1s into five peaks at 284.8, 286.3, 288.9, 289.9, and 291.4 eV (see description of the chemical states in Figure 5). The C 1s and N 1s spectra that originated from pristine g-C_3_N_4_ confirm the corresponding spectra reported for this material [34,48,49,50,51,52]. The XPS data in Table 2 clearly show that the Ti/O (0.15) and Ti/C (0.09) atomic ratios of pristine NTU-9 are slightly higher than those derived from the empirical NTU-9 formula C_24_H_6_O_18_Ti_2_ [53] (Ti/O: 0.11 and Ti/C: 0.08). This suggests that a residual Ti precursor remained on the surface of the synthesized NTU-9 MOF. The C/N ratio (0.75) of pristine g-C_3_N_4_ (Table 2) agrees well with the stoichiometric atomic composition, confirming the successful preparation of this material. The chemical compositions of the g-C_3_N_4_/NTU-9 hybrid materials, synthesized using the solvothermal method, differed significantly from those obtained by calcination in air (Table 2). The 15NTU-9/C_3_N_4__s sample, which showed the best photocatalytic activity, had the highest C/N and Ti/N ratios among all the samples analyzed. Moreover, the Ti/N ratio (0.54) of this sample was much higher than that of the corresponding calcined 15NTU-9/C_3_N_4__a sample (Ti/N = 0.10). This clearly indicates that the concentration of NTU-9 MOFs on the surface of the sample obtained by the solvothermal route was much higher than that on the sample calcined in air. The HR spectra of the elements originating from both the samples confirmed this observation (Figure 5). The chemical characteristics of the Ti 2p, O 1s, and C 1s spectra of the 15NTU-9/C_3_N_4__s sample were similar to those of NTU-9 (the corresponding Ti/O and Ti/C ratios of both materials are compared in Table 2). In addition, the intensity of the N 1s 

In addition, the intensity of the N 1s spectrum is very low because of the high surface concentration of NTU-9. The Ti 2p, O 1s, C 1s, and N 1s spectra of the calcined 15NTU-9/C_3_N_4__a sample reveal surface interactions between C_3_N_4_ and NTU-9. The Ti/O ratio (0.35) of this sample, which is higher than that of NTU-9 (0.15), indicates the partial rearrangement of the titanium composite and surface segregation of the Ti adspecies. This was confirmed by the appearance of the Ti(3+) species in the Ti 2p spectrum, along with Ti(4+) [54,55] (Figure 5). The O 1s spectrum revealed that TiO_x_ species were the main titanium chemical compounds on the surface of the calcined sample. Finally, another peak was identified in the resolved C 1s spectrum at 285.5 eV, which was attributed to the C–N bond; this bond was not detected in the pristine C_3_N_4_ or the 15NTU-9/C_3_N_4__s sample.

### 3.6. Morphology

**Figure 6:**
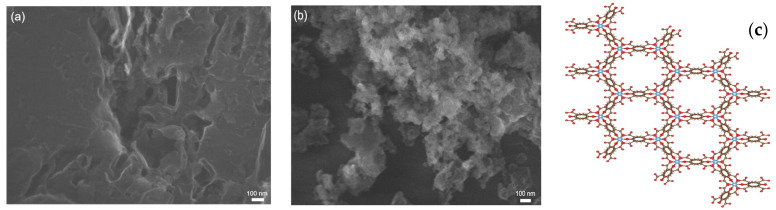
SEM images of obtained pristine materials: (**a**) g-C_3_N_4_, (**b**) NTU-9; (**c**) honey-comb like NTU-9 MOF structure.

**Figure 7:**
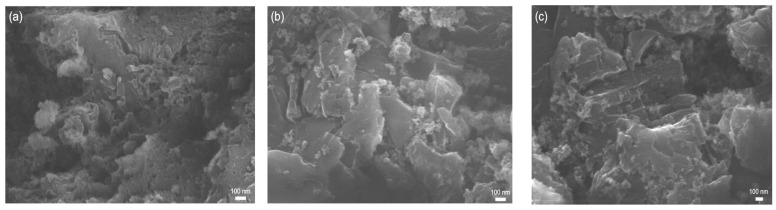

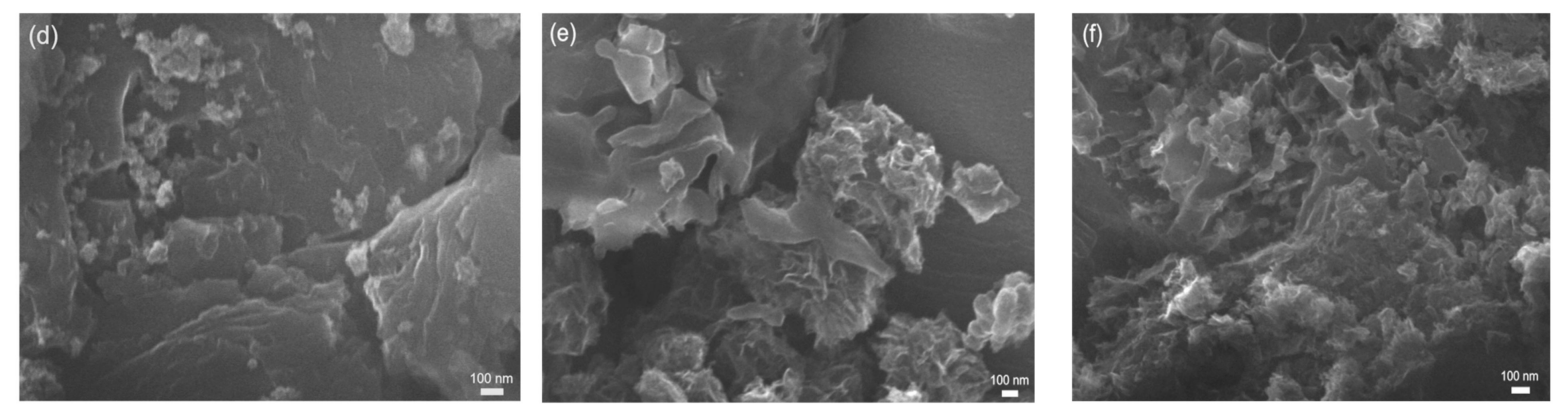
SEM images of composites obtained by: solvothermal synthesis ((**a**) 5NTU-9/C_3_N_4__s (**b**) 10NTU-9/C_3_N_4__s (**c**) 15NTU-9/C_3_N_4__s) and calcination in air ((**d**) 5NTU-9/C_3_N_4__a (**e**) 10NTU-9/C_3_N_4__a (**f**) 15NTU-9/C_3_N_4__a).

The morphologies of the obtained materials were investigated using SEM and are presented in Figure 6 and Figure 7. The SEM image of g-C_3_N_4_ (Figure 6a) shows a laminar structure with an irregular surface. The SEM image of NTU-9 (Figure 6b) shows a highly porous and irregular structure. Moreover, the structure of the honeycomb-like NTU-9 MOF was presented in Figure 6c) [53,56]. The SEM images of the composites (Figure 7) show structures originating from NTU-9 embedded in the g-C_3_N_4_ matrix. The morphology differed in appearance depending on the MOF content and composite synthesis method. The structures of the composites were irregular and indistinct, most likely owing to the presence of organic compounds. The SEM images of the samples formed by the solvothermal method (Figure 7a–c) and calcination in air (Figure 7d–f) both show a higher content of irregular particles deposited on the C_3_N_4_ surface with increasing NTU-9 content in the composites. The elements derived from NTU-9 can be clearly distinguished from those derived from g-C_3_N_4_.

### 3.7. Thermal Properties

**Figure 8:**
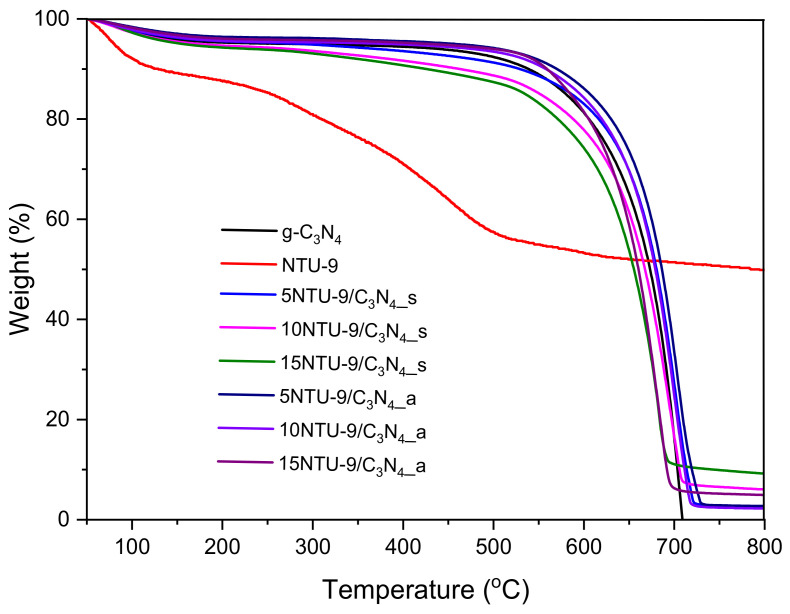
Thermogravimetric analysis of g-C_3_N_4_, NTU-9 and NTU-9/C_3_N_4_ hybrids obtained via two different routes.

Thermal properties of as-prepared g-C_3_N_4_, NTU-9 and NTU-9/C_3_N_4_ composites were investigated and TGA curves are shown in Figure 8. The weight loss step below 100 °C can be ascribed to the evaporation of adsorbed H_2_O or other solvents on the surface of all the samples. NTU-9 MOF revealed another loss step at about 250–500 °C, which was associated with the structural disintegration of the framework [57]. Graphitic carbon nitride and g-C_3_N_4_-based composites revealed very good thermal stability in the range of 100–530 °C. The decomposition of g-C_3_N_4_ occurred at about 530 °C and was completed at 710 °C due to the conversion of carbon nitride into gases containing carbon and nitrogen [58]. The NTU-9/C_3_N_4_ hybrids exhibited higher thermal stability as compared with pristine MOF, in the range of 50–650 °C. Moreover, it can be observed that thermal stability of NTU-9/C_3_N_4_ hybrids obtained by both solvothermal and calcination methods decreased with increasing content of NTU-9. The residual weight fraction of obtained hybrids was found to be in the range of 3–9% depending on the amount of NTU-9, which is considered to be the contents of titanium in the NTU-9/C_3_N_4_ hybrid materials.

### 3.8. Photocatalytic Activity

**Figure 9:**
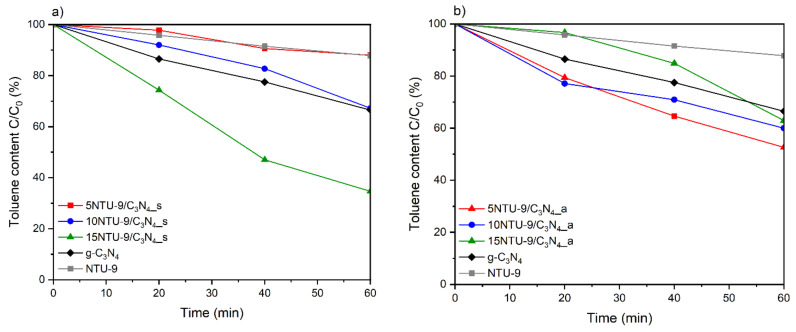
Photocatalytic activities of g-C_3_N_4_/NTU-9 composites obtained by: (**a**) solvothermal synthesis, (**b**) calcination in air.

The photocatalytic activities of the obtained materials were investigated via the photodegradation of toluene, which is a volatile organic compound (VOC) and one of the most common air pollutants originating from vehicle engines. From Figure 9, the changes in the performance of the photocatalytic reaction depending on the MOF content and preparation method of the composites can be observed. The pristine NTU-9 MOF exhibited very low photoactivity, but, when combined with g-C_3_N_4_, resulted in composites with better activity than the pristine materials. For the NTU-9/C_3_N_4_ hybrids obtained by the solvothermal method, it can be clearly seen that photoactivity increased with increasing MOF content. The best photocatalytic activity among all the prepared samples was exhibited by the 15NTU-9/C_3_N_4__s hybrid, which was obtained via the solvothermal method and contained 15 wt% NTU-9 MOFs. For this photocatalyst, the efficiency of toluene photodegradation was approximately 65% after 1 h of visible light irradiation. However, among the composites obtained by the solvothermal method, only 15NTU-9/C_3_N_4__s exhibited better activity than pristine g-C_3_N_4_. This shows that the preparation route of the NTU-9/C_3_N_4_ composites has a very strong impact on the photocatalytic activity. Moreover, the results show that a higher percentage of MOFs does not necessarily reflect an improvement in photocatalytic properties of the obtained composites; however, this was only relevant for the composites obtained using the solvothermal method. For the composites obtained by calcination in air, the photodegradation efficiency decreased with increasing NTU-9 content, but all composites exhibited better photoefficiency than unmodified C_3_N_4_ and NTU-9. This may be related to the decomposition of Ti-based MOFs during calcination in air and the formation of TiO_2_ [59], which is in good agreement with the XRD, XPS, and DRS UV-Vis analyses. FTIR spectra of the most active sample: 15NTU-9/C_3_N_4__s before and after photocatalytic process are presented in Figure S3. All the samples revealed broad absorption bands located at about 3100 cm^−1^ that can be ascribed to the stretching vibrations of O-H and N-H, suggesting the appearance of water molecules adsorbed on the surface of materials and residual amino groups attached to the edges of carbon nitride, respectively [60,61]. The several absorption peaks in the region of 1200–1640 cm^−1^ can be ascribed to the C–N and C=N stretching vibrations of the carbon nitride aromatic unit [60]. Moreover, the strong peaks at 1550 and 1422 cm^−1^ indicated the presence of carboxyl group in the framework of NTU-9 [44]. The peaks at about 888 and 806 cm^−1^ correspond to the breathing mode of triazine unites [61]. Importantly, both 15NTU-9/C_3_N_4__s samples presented similar FTIR spectra, indicating that no structural changes occurred after photocatalysis and confirming the stability of the obtained hybrid materials.

### 3.9. Proposed Photocatalytic Mechanisms

**Figure 10:**
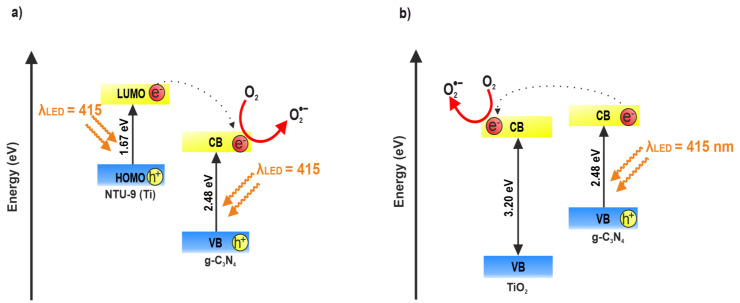
Proposed photocatalytic mechanisms: (**a**) composites obtained by solvothermal synthesis, (**b**) composites obtained by calcination in an air atmosphere.

Based on the obtained results, two possible mechanisms for the photocatalytic excitation of the composites were proposed (Figure 10). The composites obtained by solvothermal synthesis consisted of both g-C_3_N_4_ and NTU-9 components. During visible-light irradiation with a wavelength of 415 nm using an LED, excitation occurred in both NTU-9 and g-C_3_N_4_ (Figure 10a). Therefore, it was possible for electrons to be transferred between the LUMO levels of NTU-9 and the conduction band (CB) of g-C_3_N_4_, as well as between the valence band (VB) of g-C_3_N_4_ and the NTU-9 HOMO level; this is because the energy of the NTU-9 LUMO orbital is more negative than that of the g-C_3_N_4_ CB, and the position of the g-C_3_N_4_ VB is more positive than that of the NTU-9 HOMO orbital [53]. This charge transfer between bands increases the lifetime of the reactive electron–hole pairs. In the case of the composites obtained by the calcination of NTU-9 with g-C_3_N_4_ in air, TiO_2_ may have formed as a result of the decomposition of NTU-9 (Figure 10b), which is validated by the XRD, DRS, UV-Vis, and XPS analyses. Therefore, when the composite is irradiated with 415-nm LED light, only g-C_3_N_4_ (2.48 eV) is excited. Because the g-C_3_N_4_ CB is more negative [62] than the TiO_2_ CB [63], it is possible for the excited electrons to move from the CB of g-C_3_N_4_ to the CB of TiO_2_; consequently, the recombination of photoinduced carriers can be suppressed effectively. The electrons in the CB of g-C_3_N_4_ (Figure 10a) and electrons in the CB of TiO_2_-derived NTU-9 MOF (Figure 10b) are strongly reductive and may transform adsorbed molecular oxygen to produce superoxide radical anions (•O_2_^−^). Moreover, toluene adsorbed on the surface of the photocatalysts can also be directly oxidized through the holes in the HOMO of NTU-9 (Figure 10a) and holes in the VB of g-C_3_N_4_ (Figure 10b).The active oxygen species can cause mineralization and oxidize toluene into CO_2_ and H_2_O [64]. However, based on the literature data, it is known that the toluene photocatalytic degradation process is very complex and intermediate products are generated. The by-products of toluene photodegradation may include phenol, benzaldehyde, benzoic acid and formic acid, which finally decompose into carbon dioxide and water [65].

**Figure 11:**
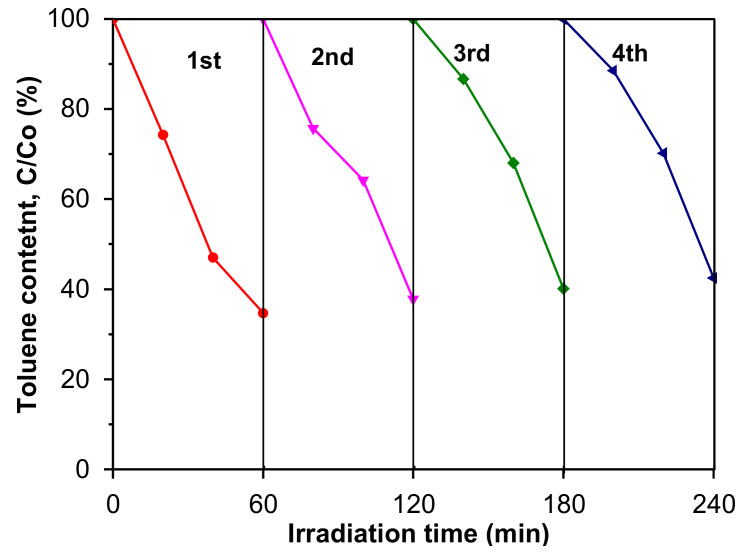
Recycling performance of 15NTU-9/C_3_N_4_ composite toward toluene photodegradation under LED light irradiation (λ_max_ = 415 nm).

Moreover, reusability is one of the most important factors in the practical applications of the photoactive materials. Therefore, the reusability of the most active sample was investigated in four recycling experiments. As shown in Figure 11, in the presence of the 15NTU-9/C_3_N_4__s hybrid material, the photocatalytic activity decreased in subsequent measurement cycles from 65% (1st cycle) to 58% (4th cycle), probably due to the blocking of the photocatalyst surface by toluene partial decomposition products. The degradation of intermediate products can compete with toluene on the surface of the photoactive sample and cause decreases in photoactivity (a reduced ability to generate reactive oxygen species).

## 4. Conclusions

In summary, a series of NTU-9/g-C_3_N_4_ hybrid materials was successfully designed and synthesized by solvothermal treatment and calcination in air. Their surface properties and photocatalytic activities toward toluene degradation under visible-light irradiation were found to be dependent on the composite preparation method and NTU-9 modifier content. Hybrid materials obtained by the solvothermal method exhibited significantly enhanced absorption properties in the visible range and lower photoluminescence intensities than pristine C_3_N_4_ and the composites prepared via calcination. The highest photoactivity among all the samples was achieved for the 15NTU-9/g-C_3_N_4__s sample, which contained 15 wt% NTU-9 and was prepared via the solvothermal method. This can be ascribed to the sample having the optimal amount of composite components, appropriate energy-level positions of NTU-9 and C_3_N_4_, enhanced charge separation, improved visible-light absorption ability, and a stable combination of the composite components. For NTU-9/g-C_3_N_4_ hybrids obtained by calcination, increasing photocatalytic activity was observed with decreasing NTU-9 content as a result of the formation of TiO_2_ from the MOFs. Two different photocatalytic mechanisms were proposed for the samples based on their synthesis method: the formation of heterojunctions between C_3_N_4_ and NTU-9 for solvothermal synthesis and between C_3_N_4_ and TiO_2_ for calcination. Our results provide a useful guide for the fabrication of efficient hybrid materials based on C_3_N_4_ and MOFs for the photocatalytic degradation of organic pollutants.

## Figures and Tables

**Table 1:** Sample label, preparation method, BET surface area, and porosity of the obtained materials.

Sample Label	NTU-9 Content (wt%)	Coupling Method	BET Surface Area (m^2^/g)	Total Pore Volume (cm^3^/g)	Average Pore Diameter (nm)
5NTU-9/C_3_N_4__s	5	solvothermal	8	0.03	12
10NTU-9/C_3_N_4__s	10	solvothermal	19	0.08	18
15NTU-9/C_3_N_4__s	15	solvothermal	14	0.05	15
5NTU-9/C_3_N_4__a	5	calcination in air	11	0.03	12
10NTU-9/C_3_N_4__a	10	calcination in air	21	0.07	13
15NTU-9/C_3_N_4__a	15	calcination in air	27	0.08	12
NTU-9	100	-	96	0.26	11
C_3_N_4_	0	-	14	0.04	14

**Table 2:** Elemental contents in the surface layer of NTU-9 and C_3_N_4_ composites and their mixtures prepared by solvothermal method (_s) and calcination under air (_a), as evaluated by XPS.

	Elemental Composition (Atomic %.)							
Sample Label	Ti	O	N	C	C/N	Ti/N	Ti/O	Ti/C
NTU-9	5.44	35.85	0	58.71	-	-	0.15	0.09
C_3_N_4_	0	0.68	56.74	42.59	0.75	0	0	0
5NTU-9/C_3_N_4__s	2.38	16.38	33.44	47.80	1.43	0.07	0.15	0.05
10NTU-9/C_3_N_4__s	3.09	18.23	28.27	50.42	1.78	0.11	0.17	0.06
15NTU-9/C_3_N_4__s	5.26	31.44	9.80	53.50	5.46	0.54	0.17	0.10
5NTU-9/C_3_N_4__a	1.09	4.60	48.75	45.56	0.93	0.02	0.24	0.02
10NTU-9/C_3_N_4__a	3.41	9.85	43.10	43.65	1.01	0.08	0.35	0.08
15NTU-9/C_3_N_4__a	4.30	12.44	41.50	41.76	1.01	0.10	0.35	0.10

## Data Availability

The data that support the findings of this study are available from the corresponding author upon reasonable request.

## Supplementary Materials

The following supporting information can be downloaded at: https://www.mdpi.com/article/10.3390/ma16145007/s1, Figure S1: Tauc plots of (a) g-C_3_N_4_ and (b) NTU-9; Figure S2: Powder XRD patterns for NTU-9/C_3_N_4_ hybrid materials; Figure S3: FTIR spectra of the most active sample: 15NTU-9/C_3_N_4__s before and after photocatalytic process; Table S1: Chemical characters of titanium and oxygen identified in Ti 2p and O1s XPS spectra, respectively, recorded for pristine NTU-9 and C_3_N_4_ composites and their mixtures prepared by solvothermal method (_s) and calcination under air (_a).
