# Supplementary material for: Design and Synthesis of NTU-9/C3N4 Photocatalysts: Effects of NTU-9 Content and Composite Preparation Method
Source: Materials (Basel). 2023 Jul 14;16(14):5007. doi: 10.3390/ma16145007 (PMC10385252; doi:10.3390/ma16145007)
Supplement: Supplementary file 1 [file materials-16-05007-s001.zip › materials-2473098-supplementary.pdf]

Supplementary Materials

Design and synthesis of NTU-9/C_3N_4 photocatalysts: effects of NTU-9 content and composite preparation method

Damian Makowski[1], Wojciech Lisowski[2], Mateusz Baluk[1], Tomasz Klimczuk[3], Beata Bajorowicz[1]

[1] Department of Environmental Technology, Faculty of Chemistry, University of Gdansk, 80-308, Gdansk, Poland

[2] Institute of Physical Chemistry, Polish Academy of Sciences, 01-224 Warsaw, Poland

[3] Department of Solid State Physics, Faculty of Applied Physics and Mathematics, Gdansk University of Technology, 80-233 Gdansk, Poland

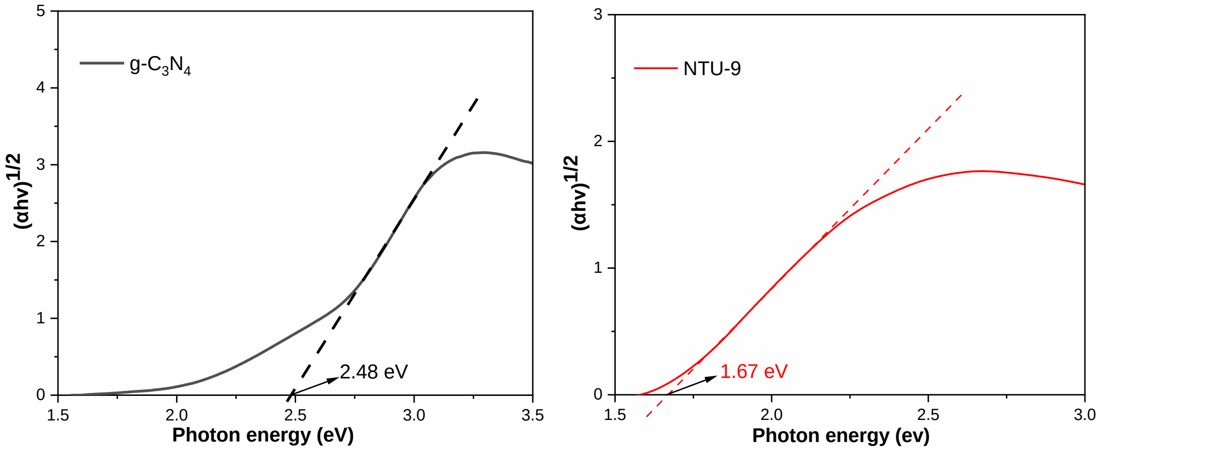

Figure S1. Tauc plots of (a) g-C_3N_4 and (b) NTU-9.

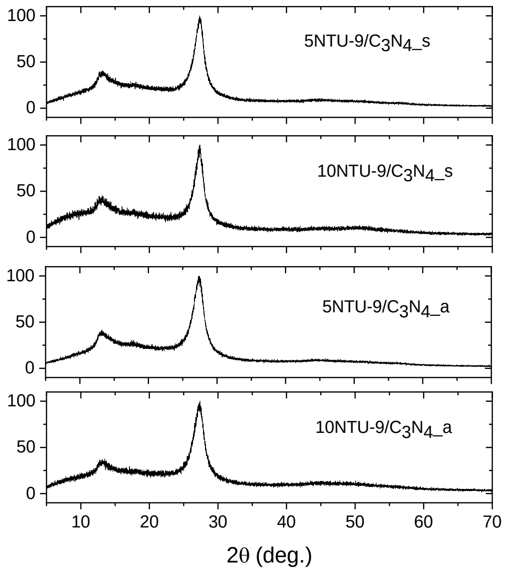

Figure S2. Powder XRD patterns for NTU-9/C_3N_4 hybrid materials.

Table S1. Chemical characters of titanium and oxygen identified in Ti 2p and O1s XPS spectra, respectively, recorded for pristine NTU-9 and C_3N_4 composites and their mixtures prepared by solvothermal method (_s) and calcination under air (_a).

	Ti $2p_{3/2}$ fraction							O 1s fraction						
Sample	Ti(4+)			Ti(3+)		Satellite (S)		A (Ti-Ox)		B (C=O)		C (-OH)		
	BE eV	FWHM eV	%	BE eV	%	BE eV	%	BE eV	(%)	BE eV	%	BE eV	%	A/B
NTU-9	459.2	1.93	95.94	0	0	462.2	4.06	530.7	15.28	532.0	74.79	533.4	9.93	0.20
C_3N_4	-	-	-	-	-	-	-	0	0	532.0	100	0	0	0.00
5NTU-9/C_3N_4_s	459.3	1.86	92.92	0	0	461.7	7.02	531.0	22.40	532.2	63.53	533.8	14.07	0.35
10NTU-9/C_3N_4_s	459.4	1.80	95.83	0	0	462.2	4.17	531.0	28.61	532.3	58.49	533.6	12.90	0.49
15NTU-9/C_3N_4_s	459.3	1.79	95.93	0	0	462.3	4.08	530.9	24.43	532.2	65.89	533.5	9.69	0.37
5NTU-9/C_3N_4_a	458.8	1.33	95.58	456.9	4.42	0	0	530.3	52.68	531.8	31.72	533.1	15.60	1.66
10NTU-9/C_3N_4_a	459.0	1.34	94.21	457.2	3.68	461.2	2.11	530.4	67.24	531.6	21.97	532.6	10.79	3.06
15NTU-9/C_3N_4_a	459.2	1.36	84.40	457.3	4.05	461.2	11.55	530.6	66.92	532.0	25.78	533.4	7.30	2.60

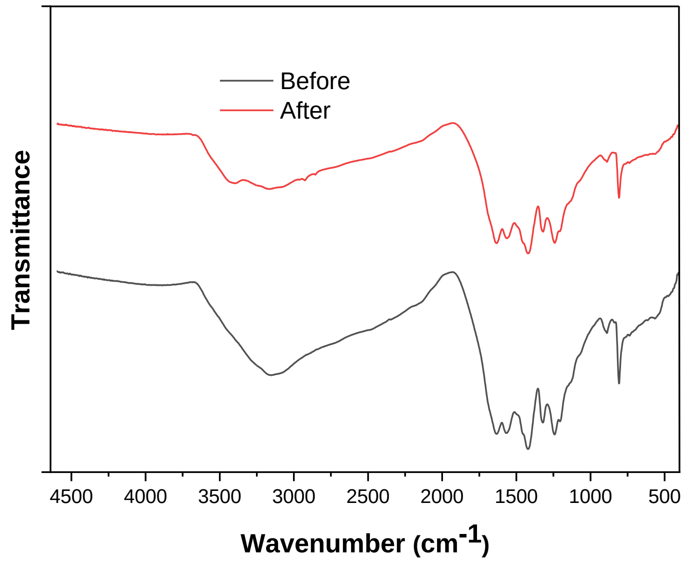

Figure S3. FTIR spectra of the most active sample: 15NTU-9/C_3N_4_s before and after photocatalytic process.
